# Correlation Analysis of Human Immunological Indicators and Nosocomial Infections, Along With Evaluation Value for Prognosis

**DOI:** 10.1155/jimr/5539590

**Published:** 2025-04-24

**Authors:** Cai-jun Wu, Jun Yan, Li-ping Sun, Lin-qin Ma, Lan Li, Jin Liu, Jia-qi Zhang, Yang Ren, Wei Bi

**Affiliations:** ^1^Department of Emergency, Beijing Dongzhimen Hospital, Beijing University of Chinese Medicine, Beijing, China; ^2^Institute of Sepsis, Beijing University of Chinese Medicine, Beijing, China; ^3^Department of Infectious Diseases, Miyun District Hospital, Beijing, China; ^4^Peking University First Hospital-Miyun Hospital, Beijing, China

**Keywords:** immune function, nosocomial infection, prognosis risk factors, T lymphocyte subsets

## Abstract

**Objective:** This study aimed to analyze the relevant risk factors for nosocomial infection (NI) in patients who were admitted to an emergency department, explore the correlation between each influencing factor and the risk of NI, and evaluate the application value of immunological indicators on the patient prognosis, all of which can provide reference for clinical guidance.

**Methods:** We prospectively enrolled 128 patients meeting the inclusion criteria who visited the emergency department of Dongzhimen Hospital, Beijing University of Chinese Medicine, from January 1 to December 31, 2019. Basic information and serum samples were collected from the patients, and flow cytometry was used. T lymphocyte subgroups, CD3^+^CD4^+^and CD3^+^CD8^+^, and natural killer (NK) cells were measured. Patients were divided into infection group and control group according to whether nosocomial infection occurred within 48 h of admission. Age, gender, type of disease, APACHE II score, Charlton score, T lymphocyte subtypes, and NK cell values were compared, and a logistic multivariate regression analysis was conducted. A multifactor regression analysis was performed on various risk factors. The nomogram website was used to draw a nomogram model of meaningful indicators, and the receiver-operating characteristic (ROC) curve was based on experimental results.

**Results:** Logistics multivariate regression analysis showed the Charlton score and NK cell count were independent risk factors for nosocomial infection. Cell counts for subsets CD3^+^CD4^+^ and CD3^+^CD8^+^ were protective factors, and the OR value and 95% CI were 5.199 (1.933–13.983), 1.248 (1.055–1.475), 0.851 (0.790–0.916), and 0.832 (0.711–0.973), *p* < 0.05. respectively. Statistical significance was set at *p* < 0.05.The nomogram model suggested that the area under the curve for predicting the risk of nosocomial infection was 0.920 (0.872–0.967), *p* < 0.001.

**Conclusion:** Patients with low CD3^+^CD4^+^ and CD3^+^CD8^+^ T lymphocyte or high NK cell count as well as high Charlton score are more likely to have nosocomial infection. Then, we speculate that the risk of nosocomial infection within 48 h is also high for patients with underlying diseases and immune function that is affected and suppressed on admission, regardless of whether infection occurs during hospitalization.

## 1. Introduction

The term nosocomial infection (NI) refers to an infection that does not exist or has an incubation period at the time of patient admission. It is a common adverse event during hospitalization. Because some diseases are easily transmitted and infected in hospitals, including catheter-associated urinary tract infections, centerline-associated bloodstream infections, surgical site infections, ventilator-associated pneumonia, hospital-acquired pneumonia, and *Clostridioides difficile* infections [1, 2]. The most common types of NIs include urinary tract infection (UTI), pneumonia (PNA), and bacteremia, which together account for almost half of all NIs [3]. Infected patients experience prolonged hospitalization, other complications, a poor prognosis, permanent disability, or even death. Patients in the emergency department have complex diseases or critical health conditions and relatively poor immunity, and they require more invasive monitoring. Hence, the possibility of NI is elevated [4, 5]. It is imperative to investigate the risk factors for nosocomial infections in emergency department patients to prevent or manage such infections.

Recent studies have demonstrated the pivotal role of the innate immune system in determining infection outcomes, with impaired immune function closely associated with the heightened risk and adverse consequences of nosocomial infection in critically ill patients [6]. It is well-established that lymphocytes are crucial for inducing cellular immune responses, and dysfunction of lymphocyte subsets can result in immune function abnormalities. As a type of lymphocytes, changes in NK cell levels can also serve as an indicator of immune function abnormalities [7]. Recent research has indicated that the CD4/CD8 ratio can predict the occurrence of surgical site infection in fractures with impaired immune function and is correlated with the incidence of nosocomial infection [8, 9].

In *Acinetobacter Baumannii* infection [10], CD4+ T cell ratio increased and NK cell activity decreased. NK cells were associated with immune tolerance in sepsis patients. In infectious diseases, the count of NK cells increases while CD3^+^ and CD4^+^ T cells decrease, with more pronounced changes observed in severe and acute stages. These alterations may be linked to the significantly impaired immune function of patients during severe and acute stages of infection [7]. In conclusion, immune indeces may assist emergency physicians in predicting the occurrence of nosocomial infections and assessing the severity of patients with nosocomial infections. Current studies on the correlation between human immune indicators and nosocomial infection primarily focus on a single type of immune cells. This paper aims to analyze the clinical utility of multiple immunological indicators and related scores for patient prognosis, including T lymphocyte subtype, NK cell value, APACHE II score, and Charlson score, in order to provide guidance for clinical practice.

## 2. Materials and Methods

### 2.1. Inclusion and Exclusion Criteria

Patients hospitalized at the emergency Department of Dongzhimen Hospital, Beijing University of Chinese Medicine, from January 1 to December 31, 2019, were prospectively included. Patients who developed nosocomial infections during hospitalization were assigned to the infected group, while those who did not develop any infections either before or during hospitalization were placed in the noninfected group. NI diagnosed according to the standards for NI surveillance issued by the Ministry of Health of China [11]. When the incubation period of an infection is not clearly defined, if the patient develops symptoms such as fever, cough, expectoration, abdominal pain, diarrhea, frequent urination, and urgent urination 48 h after admission, accompanied by abnormal laboratory findings at the corresponding sites (such as elevated blood leukocyte count, increased urine leukocytes, etc.) and positive etiological test results (such as pathogens cultured from specimens like sputum, urine, and blood), it can be diagnosed as a nosocomial infection. For an infection with a well-defined incubation period, if the onset occurs after the average incubation period from the time of admission, it can also be determined as a nosocomial infection. The inclusion criteria were: (1) no infection occurred before and within 48 h of admission, (2) the duration of hospitalization was more than 48 h, and (3) the patient must be 18 years old or older. The exclusion criteria were (1) malignant tumor, severe immunodeficiency (T-cell count less than 0.05 × 10^9^/L unless maternal T cells are present) [12], or application of immunosuppressants; (2) serious primary heart (New York Heart Association class III–IV), liver (ALT/AST/ALP > 5x upper limit of normal range (ULN)] or TBIL > 3x ULN or CTP score ≥ 10), lung conditions (acute respiratory failure, acute respiratory syndrome, etc.), kidney impairment (eGFR calculated through the CKD-EPI equation <30 mL /min/1.73 m^2^), or hematopoietic system diseases (acute leukemia, myelodysplastic syndromes, etc.); [13–15] (3) patient is age 90 years old or older. The Dongzhimen Hospital, Beijing University of Chinese Medicine Ethics Committee, reviewed and approved this study.

### 2.2. General Information

The same group of doctors collected participants' detailed medical histories and related data, including (1) general demographic information, such as name, age, sex, past medical history, infections position, illness severity, and comorbidities on admission. Illness severity was evaluated by Acute Physiology and Chronic Health Evaluation (APACHE) II score during the first 24 h of admission and the Charlton score was calculated. (2) Hospitalization days and discharge condition outcome48 h after admission were assessed. The clinical protocol for this study was approved by the hospital's ethics committee.

### 2.3. Detection and Statistical Analysis

The laboratory center of the hospital carried out all the tests within 24 h of admission. The lymphocyte subtypes and counts were determined at the time of admission. The lymphocyte subsets were identified using the following monoclonal antibodies: anti-CD45-PerCP, anti-CD3-FITC, anti-CD8-PE, anti-CD4-APC, and anti-CD16/56-PE. With the complete blood analysis (Becton, Dickinson and Company, Franklin Lakes, NJ, United States), T lymphocytes and NK cells were analyzed by fluorescence-activated cell sorter (FACS) according to the standard scheme. The number of positive cells was analyzed, including the expression of CD3^+^CD4^+^, CD3^+^CD8^+^, and T lymphocytes, as well as the percentage of NK cells in the lymphocyte door. SPSS 25.0 statistical software (IBM Corp., Armonk, NY, United States) was used for the statistical analysis of experimental data. If the measured data is normal and homogeneous in variance, they are reported as mean plus or minus standard deviation (mean ± SD). An independent sample *t*-test compared the differences between the two groups. The counting data were arranged in the format of frequency (percentage). Differences between the two groups were compared with the chi-square test. The stepwise logistic regression model was used in multivariate analysis, and a nomogram website (http://111.229.212.9:3838/inomogram) was used to draw a nomogram model for predicting NI risk. The inspection level was *α* = 0.05.

## 3. Results

### 3.1. Comparison of Clinical Data Between Two Groups

**Figure 1:**
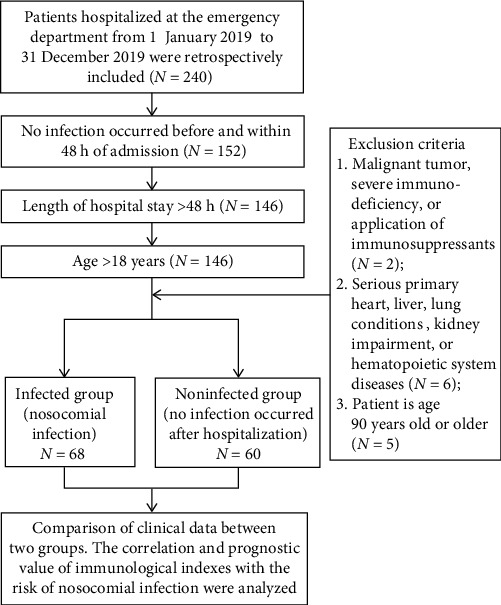
Flow chart of analysis population.

Out of the 128 patients included in this study, 68 (53.13%) contracted nosocomial infection (Figure 1).Among the infected group, patients were mainly respiratory system infection (57.35%), followed by urinary system infection (26.47%), as shown in Table 1. The baseline characteristics and risk factors between nosocomial infection patients and the control group were compared. The results showed that cell counts of the T lymphocyte subsets CD3^+^CD4^+^ and CD3^+^CD8^+^ were lower in the NI group than the uninfected group. NK cell counts, Charlton scores, death patients, cerebrovascular disease patients, and length of hospital stay were higher in the NI group than the uninfected group (*p* < 0.05). Gender, age, comorbidities, and APACHE II score showed no significant differences (*p* > 0.05). See Table 2 for details.

### 3.2. Logistic Multivariate Regression Analysis of Nosocomial Infection in Hospitalized Patients

A logistic multivariate regression analysis was conducted with the statistically significant variables from the univariate analysis, with nosocomial infection as the dependent variable (1 = occurred, 0 = did not occur). The results showed that Charlton score and NK cell count were independent risk factors for nosocomial infection, and their OR and 95% CI were 5.199 (1.933 −13.983) and 1.248 (1.055–1.475), respectively, *p* < 0.05. The CD3^+^CD4^+^ and CD3^+^CD8^+^ cells were protective factors against nosocomial infection in patients, and their OR values and 95% CI were 0.851 (0.790–0.916) and 0.832 (0.711–0.973), respectively, *p* < 0.05 (see Table 3).

### 3.3. Establishment of Nomogram Model to Predict Individual Risk of NI

**Figure 2:**
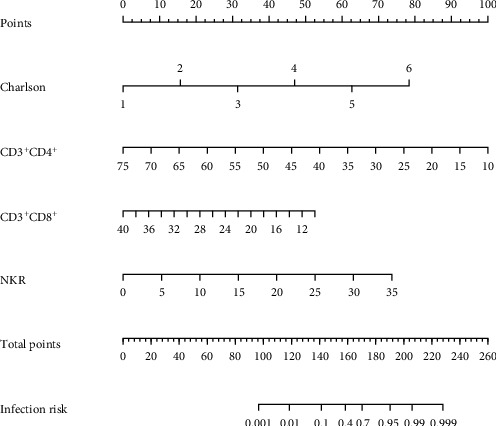
A nomogram model for predicting the occurrence of nosocomial infections.

The nomogram model for NI risk prediction was constructed according to the logistic regression analysis results, as shown in Figure 2. See Table 4 for a detailed correspondence scale for the prediction of specific hazards.

### 3.4. ROC Curve Analysis of T Lymphocyte Subtypes for Predicting NI

**Figure 3:**
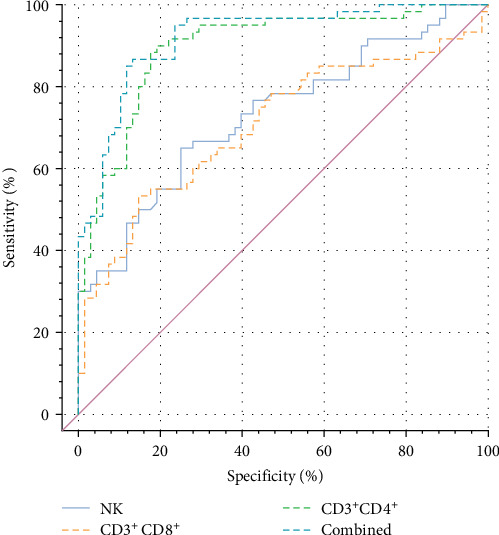
ROC curve analysis of T lymphocyte subtypes for predicting NI. ROC, receiver-operating characteristic.

Receiver-operating characteristic (ROC) curve analysis of CD3^+^CD4^+^ and CD3^+^CD8^+^ T lymphocyte showed that the AUC of CD3^+^CD4^+^ cells to predict the risk of NI was 0.898 (0.842–0.953). The AUC of CD3^+^CD8^+^ cells to predict the risk of NI was 0.706 (0.614–0.799). The AUC of NK cells to predict the risk of NI was 0.732 (0.645–0.820), and the AUC to predict the risk of NI was 0.920 (0.872–0.967) after the combination of all three cells. which demonstrates that the combined prediction accuracy of the three methods is high and has a great reference value. See Figure 3 for details.

## 4. Discussion

This study explored the related risk factors of nosocomial infection and the predictive value of human immunology-related indicators such as T lymphocyte subtypes CD3^+^CD4^+^ and CD3^+^CD8^+^ and NK cells (natural killer cells) on the occurrence of nosocomial infection. It was found that the immunology-related indicators measured in this study can predict the possibility of nosocomial infection in hospitalized patients.

Under normal circumstances, the number of lymphocytes and their subtypes in peripheral blood is relatively stable, which means it can generate a reasonable immune response and eliminate foreign antigens without damaging itself. However, during hospitalization, when there are high-risk factors for nosocomial infection, the balance will be broken under the condition of poor autoimmune function, which will lead to changes in lymphocyte subtypes and NK cell numbers in the peripheral blood. This study found that Charlton score and NK cell count are independent risk factors for nosocomial infection. The T lymphocyte subsets (CD3^+^CD4^+^ and CD3^+^CD8^+^) cell counts are protective factors. The levels of CD3^+^CD4^+^ and CD3^+^CD8^+^ cells in the peripheral blood of nosocomial infection patients decreased, indicating that the number of active cells was insufficient to participate in the cellular immune response. The decreased levels of CD3^+^CD4^+^ and CD3^+^CD8^+^ T lymphocyte subsets indicate that the cellular immune function of nosocomial infection patients is low. Under the influence of the surrounding microenvironment, these cells can be polarized into different subtypes (including Th1, Th2, Th17, and Treg cell phenotypes) [16]. These T cell subtypes will produce cytokines to regulate the surrounding inflammatory cells. Th1 cells, for example, are a good source of IFN-*γ*, Th17 cells secrete the proinflammatory cytokine IL-17, while Treg cells inhibit cellular immune response [17], and IFN-*γ* can cause lymphocyte apoptosis [18]. One study found that CD3^+^CD4^+^ T lymphocytes decreased quickly when the patient had severe acute respiratory syndrome (ARDS), resulting in the impairment of cellular immune function [19]. The decreased levels of CD3^+^ T cells and CD4^+^ T cells indicate that the immune function is low, and the patient's condition is critical, so the immune cells cannot generate an effective immune response to pathogens [20]. Reduced CD3^+^CD4^+^ T cells also reduces the ability to clean up pathogens [21, 22]. It can be inferred that the complex immune system leads to the damage of T lymphocytes and the weakening of immune response ability during nosocomial infection. The research data found that activated T cells play a vital role in the pathophysiology of nosocomial infection because the main proportion of lymphocytes in peripheral blood changes [9]. However, for the uninfected group, the activation of these T lymphocytes will be relatively complex if many basic diseases are persistent [23].

A logistic multivariate regression analysis found that the Charlton score and NK cell count of nosocomial infection patients were higher than those of the uninfected group. This was a high-risk factor for nosocomial infection compared to T cells that were the protective factors. Patients with basic diseases often have poor prognosis, longer hospitalization duration, and higher hospitalization cost. NK cells play a crucial role in the early control of viral infection [24]. As a component of natural immune defense, it has been demonstrated in both humans and mice that these cells are capable of directly promoting cellular immunity and exerting immunomodulatory functions through the secretion of cytokines, such as IFN-*γ*, TNF-*α*, IL-10, and G-CSF [7]. Their direct release of cytotoxicity or various inflammatory cytokines and chemokines may result in tissue damage and disease exacerbation [25], thereby highlighting their significant involvement in the pathogenesis of nosocomial infections.

This study confirms that evaluating immune function of patients in time after admission by scoring Charlton and monitoring T lymphocyte subtypes could effectively determine whether the patients may develop nosocomial infection. In the course of medical treatment, we should concentrate on improving the diagnosis management of nosocomial infection, on deterring disease transmission, spread, and infection, as well as, focusing on improving the immune system to prevent nosocomial infection, which has finally proven to be a worthwhile effort. In other word, this study shows that regardless of whether there is infection during hospitalization or not, the risk of nosocomial infection after 48 h is also very high for patients with primary diseases and immune function affected and inhibited at admission. The Charlton score and the monitoring of human immune indexes have predictive value for the occurrence of nosocomial infection and have important clinical significance for the prognosis of patients.

Previous research has indicated that immunosuppression is a risk factor for nosocomial infection; however, there is limited literature on the association between immune indicators and the prognosis of nosocomial infection, especially in emergency department patients with a higher probability of nosocomial infection [26, 27]. The novelty of this study lies in its exploration of the predictive value of immune indicators for nosocomial infection in emergency department patients. For the first time, we have integrated T lymphocyte subsets and NK cells to forecast the risk of nosocomial infection and develop a nomogram model. One limitation of this study is the lack of grouping and analysis of different types of nosocomial infections, potentially restricting the applicability of the prediction model to specific disease types or bacteria. The observed phenomenon in this study may be influenced by the ability of microorganisms to regulate or stimulate specific categories of immune cells rather than being solely attributed to their intrinsic value.

## Figures and Tables

**Table 1:** Distribution of infection sites in 68 patients with nosocomial infection.

Infections position	Number of cases	Ratio (%)
Lung	39	57.35
Coeliac cavity	18	26.47
Nervous system	7	10.29
Other	4	5.88
Total	68	100.00

**Table 2:** Comparison of the baseline characteristics and risk factors for the infected group and the noninfected group.

Characteristics and clinical data	Non-infected (*n* = 60)				Infected (*n* = 68)	t/*χ*^2^	*p*-Value
Demographic characteristics							
Sex	—				—	0.008	0.929
Male	34				38	—	—
Female	26				30	—	—
Age (*x* ± *s*, years)	52.7 ± 16.49				52.38 ± 18.72	0.101	0.920
Comorbidities—*n* (%)							
Hypertension	45				44	1.594	0.207
Diabetes mellitus	20				21	0.088	0.767
Cardiopathy	22				21	0.478	0.489
Nephropathy	5				7	0.144	0.704
Cerebrovascular disease	11				30	9.734	0.002
Hepatopathy	5				1	3.36	0.067
Illness severity							
APACHE II (*x* ± *s*, points)	20.87 ± 4.50				19.99 ± 4.05	0.921	0.359
Charlton score (*x* ± s, minute)	1.75 ± 0.75				2.96 ± 1.25	6.698	< 0.001
Laboratorial results ± SD							
CD3^+^CD4^+^ (%)	47.23 ± 11.57				28.60 ± 9.47	10.015	< 0.001
CD3^+^CD8^+^ (%)	24.46 ± 5.46				21.25 ± 2.83	4.100	< 0.001
NK cells	14.78 ± 6.65				20.20 ± 5.04	5.153	< 0.001
Survival outcome	—				—	12.887	<0.001
Survival patients	57				48	—	—
Death patients	3				20	—	—
Length of hospital stay	12.17 ± 5.34				22.76 ± 14.41	28.009	<0.001

**Table 3:** Analysis of risk factors for nosocomial infection.

	B	SE	Wald	*p*-Value	OR	Upper limit of 95% OR	95% OR lower limit
Charlton score	1.648	0.505	10.663	0.001	5.199	1.933	13.983
CD3^+^CD4^+^	−0.162	0.038	18.590	<0.001	0.851	0.790	0.916
CD3^+^CD8^+^	−0.184	0.080	5.268	0.022	0.832	0.711	0.973
NK cells	0.221	0.086	6.687	0.010	1.248	1.055	1.475
Constant	6.345	2.922	4.714	0.030	—	—	—

**Table 4:** The nomogram correspondence scale for predicting the occurrence of nosocomial infection.

Charlton score	1	2	3	4	5	6	—	—	—	—	—	—	—	—
Points	0	16	31	47	63	78	—	—	—	—	—	—	—	—

CD3^+^CD4^+^	10	15	20	25	30	35	40	45	50	55	60	65	70	75
Points	100	92	85	77	69	62	54	46	38	31	23	15	8	0

CD3^+^CD8^+^	10	12	14	16	18	20	22	24	26	28	30	32	34	36
Points	53	49	46	42	39	35	32	28	25	21	18	14	11	7

NK	0	5	10	15	20	25	30	35	—	—	—	—	—	—
Points	0	11	21	32	42	53	63	74	—	—	—	—	—	—

Total points	97	119	141	158	170	190	206	228	—	—	—	—	—	—
Risk	0.001	0.010	0.100	0.400	0.700	0.950	0.990	0.999	—	—	—	—	—	—

## Data Availability

All data generated or analyzed during this study are included in this published article.

## References

[1] Monegro A. F., Muppidi V., Regunath H. (2025). Hospital-Acquired Infections. *StatPearls [Internet]*.

[2] Guo S. J., Ren S., Xie Y. E. (2018). Evaluation of the Protective Efficacy of a Fused OmpK/Omp22 Protein Vaccine Candidate against Acinetobacter Baumannii Infection in Mice. *Biomedical and Environmental Sciences: BES*.

[3] Magill S. S., Edwards J. R., Bamberg W. (2014). Multistate Point-Prevalence Survey of Health Care-Associated Infections. *The New England Journal of Medicine*.

[4] Lewis S. R., Schofield-Robinson O. J., Rhodes S., Smith A. F. (2019). Chlorhexidine Bathing of the Critically ill for the Prevention of Hospital-Acquired Infection. *Cochrane Database of Systematic Reviews*.

[5] Liu X. Y., Xue K. N., Rong R., Zhao C. H. (2016). Fault Tree Analysis: Investigation of Epidemic Hemorrhagic Fever Infection Acquired in Animal Laboratories in China. *Biomedical and Environmental Sciences*.

[6] Duggal N. A., Snelson C., Shaheen U., Pearce V., Lord J. M. (2018). Innate and Adaptive Immune Dysregulation In Critically Ill ICU Patients. *Scientific Reports*.

[7] Sun L. P., Hu Y. J., Niyonsaba A. (2014). Detection and Evaluation of Immunofunction of Patients With Severe Fever With Thrombocytopenia Syndrome. *Clinical and Experimental Medicine*.

[8] Liu B., Li K. P., Li S. T., Zhao R. G., Zhang Q. (2023). The Association Between the CD4/CD8 Ratio and Surgical Site Infection Risk Among HIV-Positive Adults: Insights From a China Hospital. *Frontiers in Immunology*.

[9] Morris A. C., Anderson N., Brittan M. (2013). Combined Dysfunctions of Immune Cells Predict Nosocomial Infection in Critically Ill Patients. *British Journal of Anaesthesia*.

[10] Shi J., Sun T., Cui Y. (2020). Multidrug Resistant and Extensively Drug Resistant Acinetobacter Baumannii Hospital Infection Associated With High Mortality: A Retrospective Study in the Pediatric Intensive Care Unit. *BMC Infectious Diseases*.

[11] Wang Y. H., Ren J., Yao Z. Q. (2023). Clinical Impact and Risk Factors of Intensive Care Unit-Acquired Nosocomial Infection: A Propensity Score-Matching Study From 2018 to 2020 in a Teaching Hospital in China. *Infection and Drug Resistance*.

[12] Dvorak C. C., Haddad E., Heimall J. (2023). The Diagnosis of Severe Combined Immunodeficiency: Implementation of the PIDTC 2022 Definitions. *Journal of Allergy and Clinical Immunology*.

[13] Wuethrich P. Y., Studer U. E., Thalmann G. N., Burkhard F. C. (2014). Intraoperative Continuous Norepinephrine Infusion Combined With Restrictive Deferred Hydration Significantly Reduces the Need for Blood Transfusion in Patients Undergoing Open Radical Cystectomy: Results of a Prospective Randomised Trial. *European Urology*.

[14] Sánchez-Sánchez C., Palacios-Guillén M., Rivero-Robles L., Cuentas-Vela J., Céspedes-Valdez M., Hernández-Obando E. (2022). Diaphragmatic Hernia in a Chronic Ambulatory Peritoneal Dialysis Patient. *Revista De Nefrologia Dialisis Y Trasplante*.

[15] Arabi Y. M., Fowler R., Hayden F. G. (2020). Critical Care Management of Adults With Community-Acquired Severe Respiratory Viral Infection. *Intensive Care Medicine*.

[16] Ren J., Crowley S. D. (2019). Role of T-Cell Activation in Salt-Sensitive Hypertension. *American Journal of Physiology - Heart and Circulatory Physiology*.

[17] Zhang J. D., Crowley S. D. (2015). Role of T Lymphocytes in Hypertension. *Current Opinion in Pharmacology*.

[18] Sobek V., Balkow S., Körner H., Simon M. M. (2002). Antigen-Induced Cell Death of T Effector Cells In Vitro Proceeds via the fas Pathway, Requires Endogenous Interferon-Gamma and is Independent of Perforin and Granzymes. *European Journal of Immunology*.

[19] Li T., Qiu Z., Han Y. (2003). Rapid Loss of Both CD4+ and CD8+ T Lymphocyte Subsets During the Acute Phase of Severe Acute Respiratory Syndrome. *Chinese Medical Journal*.

[20] Roger P. M., Hyvernat H., Ticchioni M., Kumar G., Dellamonica J., Bernardin G. (2012). The Early Phase of Human Sepsis is Characterized by a Combination of Apoptosis and Proliferation of T Cells. *Journal of Critical Care*.

[21] Zhang X., Wang X., Qin L. (2023). Changing Roles of CD3 + CD8 Low T Cells in Combating HIV-1 Infection. *Chinese Medical Journal*.

[22] Reina-Campos M., Scharping N. E., Goldrath A. W. (2021). CD8+ T Cell Metabolism in Infection and Cancer. *Nature Reviews Immunology*.

[23] Citro A., Barnaba V., Martini H. (2014). From T Cell Apoptosis to Chronic Immune Activation in Inflammatory Diseases. *International Archives of Allergy and Immunology*.

[24] Lee S. H., Miyagi T., Biron C. A. (2007). Keeping NK Cells in Highly Regulated Antiviral Warfare. *Trends in Immunology*.

[25] Maghazachi A. A. (2010). Role of Chemokines in the Biology of Natural Killer Cells. *Current Topics in Microbiology and Immunology*.

[26] Adrie C., Lugosi M., Sonneville R. (2017). Persistent Lymphopenia Is a Risk Factor for ICU-Acquired Infections and for Death in ICU Patients With Sustained Hypotension at Admission. *Annals of Intensive Care*.

[27] Kreitmann L., Helms J., Martin-Loeches I. (2024). ICU-Acquired Infections in Immunocompromised Patients. *Intensive Care Medicine*.

